# Supplementary material for: Perturbation of METTL1-mediated tRNA N7- methylguanosine modification induces senescence and aging
Source: Nat Commun. 2024 Jul 8;15:5713. doi: 10.1038/s41467-024-49796-8 (PMC11231295; doi:10.1038/s41467-024-49796-8)
Supplement: Supplementary file 1 — Supplementary Information [file 41467_2024_49796_MOESM1_ESM.pdf]

Perturbation of METTL1-Mediated tRNA N^7- Methylguanosine Modification Induces Senescence and Aging

Yudong Fu[1,2,3,4#], Fan Jiang[2,3,4#], Xiao Zhang[1,2,3,4], Yingyi Pan[5], Rui Xu[6], Xiu Liang[2,3,4], Xiaofen Wu[2,3,4], Xingqiang Li[7], Kaixuan Lin[2,3,4], Ruona Shi[2,3,4], Xiaofei Zhang[1,2,3,4], Dominique Ferrandon[5,8,9], Jing Liu[1,2,3,4,10], Duanqing Pei[11], Jie Wang[1,2,3,4,10*], Tao Wang[1,2,3,4*]

[1]University of Chinese Academy of Sciences, Beijing, China. [2]Guangdong Provincial Key Laboratory of Stem Cell and Regenerative Medicine, Guangdong-Hong Kong Joint Laboratory for Stem Cell and Regenerative Medicine, Guangzhou Institutes of Biomedicine and Health, Chinese Academy of Sciences, Guangzhou, China. [3]GIBH-HKU Guangdong-Hong Kong Stem Cell and Regenerative Medicine Research Centre, Guangzhou, China. [4]GIBH-CUHK Joint Research Laboratory on Stem Cell and Regenerative Medicine, Guangzhou, China. [5]Sino-French Hoffmann Institute, Guangzhou Medical University, Guangzhou, China. [6]Department of pediatrics, Foshan maternal and children's hospital affiliated to southern medical university, Foshan, Guangdong 528000. [7]Bioland Laboratory, Guangzhou, China. [8]Université de Strasbourg, Strasbourg, France.[9]Modèles Insectes de l'Immunité Innée, UPR 9022 du CNRS, Strasbourg, France. [10]Joint School of Lifesciences, Guangzhou Institutes of Biomedicine and Health, Chinese Academy of Sciences, Guangzhou, 510530, China, Guangzhou Medical University, Guangzhou, 511436, China.[11]School of Life Sciences, Westlake University, Hangzhou, China. #These authors contributed equally. *Corresponding authors, wang_jie01@gibh.ac.cn; wang_tao@gibh.ac.cn

FigureS1

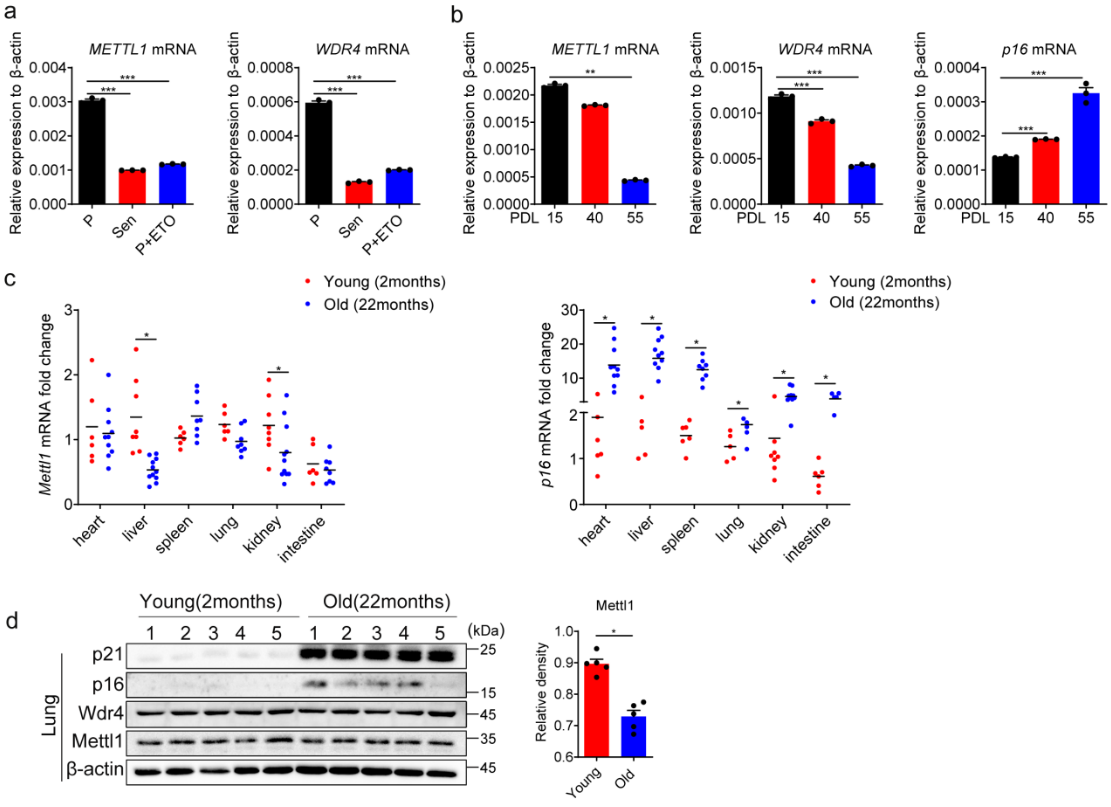

FigS1. Measurement of METTL1/WDR4 level during senescence and aging.

a METTL1 and WDR4 level were detected in senescence models. P represents proliferating IMR90 cells. Sen refers to senescent IMR90 cells. P+ETO stands for proliferating cells treated with ETO. **b** Transcript levels of METTL1, WDR4, and senescence markers were measured at various time points during senescence using RT-qPCR. **c** Transcription levels of Mettl1 and p16 were shown in tissues of young and old mice, n=8 mice per group (4 female and 4 male mice).**d** Protein level of Mettl1 and Wdr4 were shown in lung tissues of young and old mice, n=5 mice per group (2 female and 3 male mice). Data in panel **a, b** and **c** is presented as mean ± SEM, ***$p < 0.001$, *$p < 0.05$, unless otherwise specified, n = 3. P value was calculated by two sides student's T test. Source data are provided as a Source Data file.

FigureS2

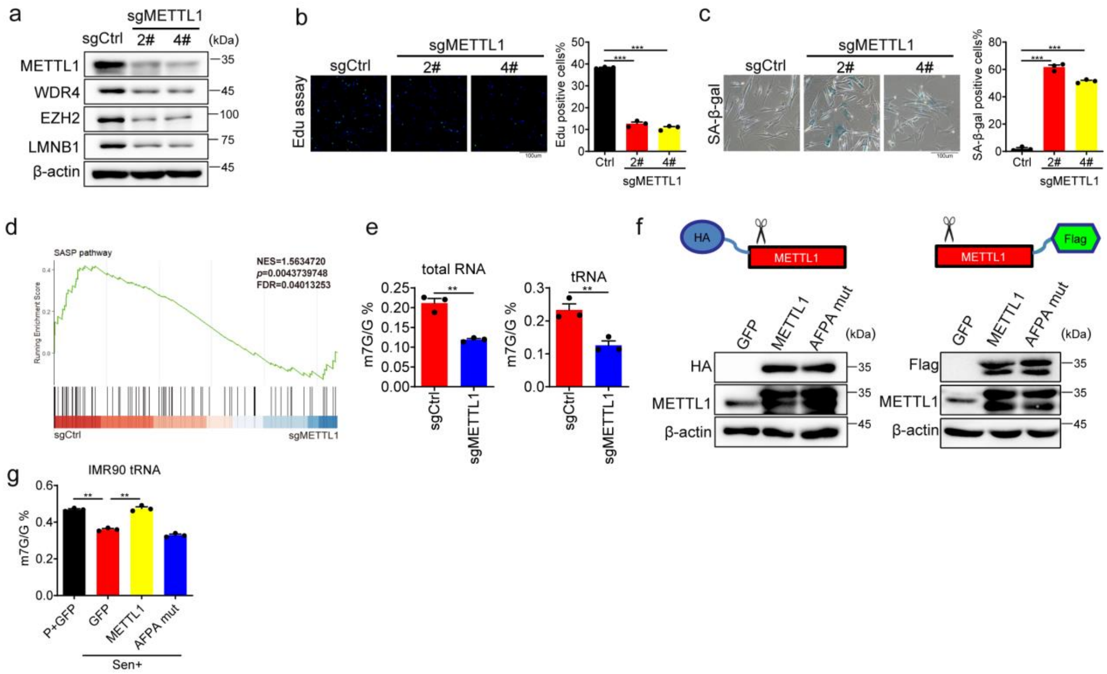

FigS2.METTL1 Deficiency Accelerate Cell Senescence

a METTL1 was depleted by CRISPR in IMR90 cells, senescence status was assessed by assays including western blotting(**a**), SA-β-gal staining(**b**), EdU incorporation(**c**). Scale bar = 100 μm, P values were calculated using a one-tailed Student's t-test. **d** Differentially regulated SASP genes in RNA-Seq of METTL1 knockout cells were enriched using GSEA analysis. **e** m7G levels in total RNA and tRNA from METTL1 KO cells were analyzed using HPLC/MS. Three repeats were conducted. **f** Ectopic expression of METTL1 and AFPA-mut proteins, labeled with a FLAG tag at either the C-terminus or HA at the N-terminus, were detected in IMR90 cells with anti-METTL1 or tag antibodies as indicated. The left panel displays Western blotting using METTL1-FLAG construct; the right panel exhibited Western blotting using HA-METTL1 construct. **g** m7G modification in IMR90 cells with ectopic expression of METTL1 and AFPA-mut was analyzed using HPLC-MS. Source data are provided as a Source Data file.

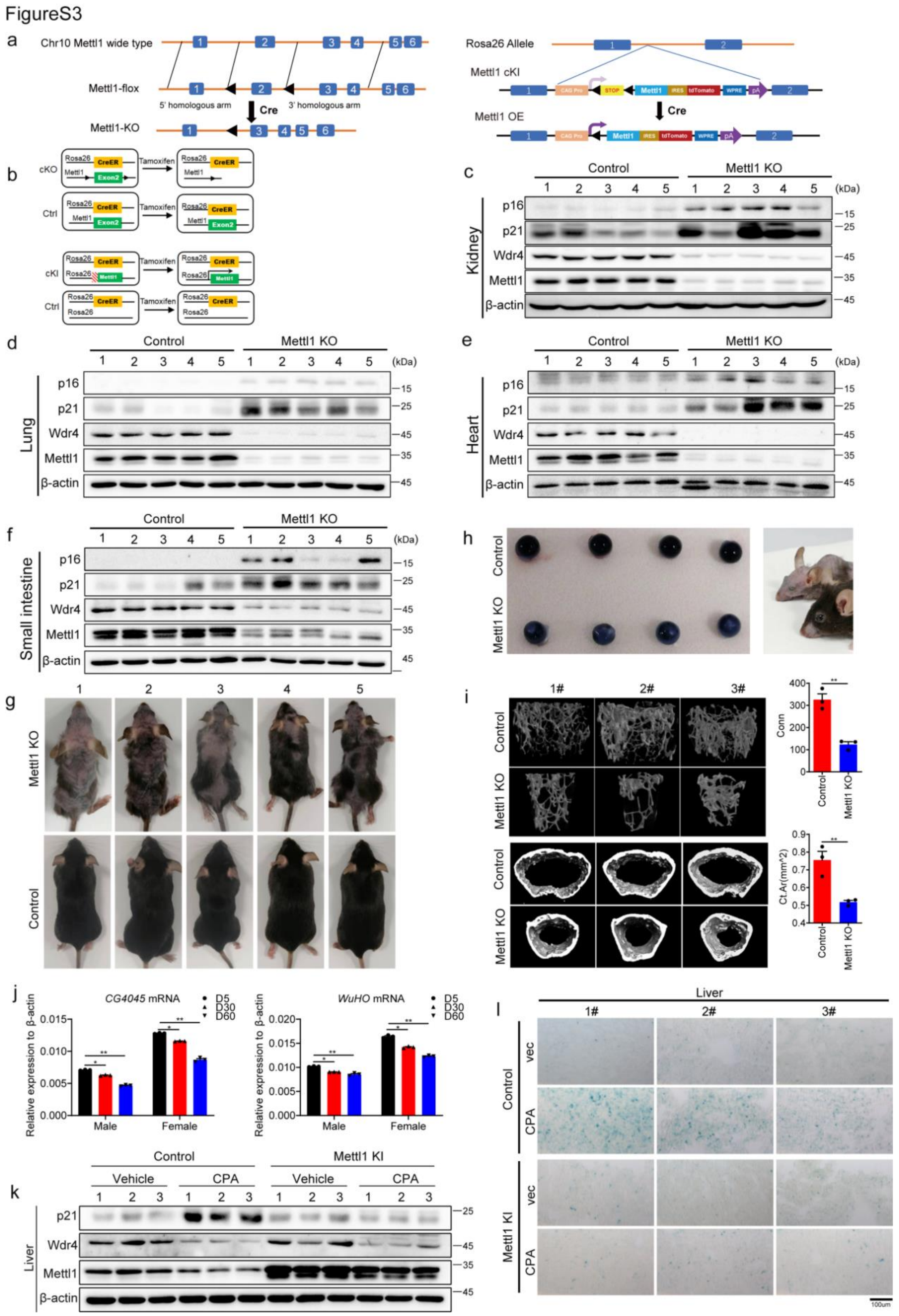

FigS3. Depletion of Mettl1 Induces Premature Aging

a Schematic diagram illustrating the construction strategy for conditional METTL1 knockout and conditional METTL1 knockin mice(**a**) and the experimental strategy for METTL1 knockout and conditional METTL1

overexpression using Mettl1flox/flox, Rosa26$^{LSL-Mettl1}$, and Rosa26^{CreERT2} mice(**b**). Western blotting for p16 and p21 in Mettl1 KO mice were assessed using kidney(**c)**, lung (**d**), heart(**e**) and small intestine tissues(**f**), n=5 mice per group (3 female and 2 male). **g** Representative photos of tamoxifen-administered control and knockout mice at 6 months of age. **h** Pictures of eyeballs from Mettl1 knockout (KO) and control mice were taken to calculate the percentage of cataracts. **i** Mettl1 knockout (KO) and control mice were euthanized at around 6 months of age to take bones for 3D scanning. Cortical bones from control and Mettl1 KO mice were calculate the volume and thickness. n=3 mice per group (2 female and 1 male). **j** CG4045 and Wuho transcripts were determined by RT-qPCR using Day5, Day30 and Day60 Drosophila. **k** Detection of the Mettl1, Wdr4 proteins and p21 protein in liver with CPA or vehicle treatment as indicated. Representative result was shown, n=3 mice per group (2 female and 1 male). **l** SA-β-gal staining was performed on liver sections from the CPA model, n=3 mice per group (2 female and 1 male). Unless otherwise indicated, *in vitro* assays were biologically repeated three times. All data were presented as the mean ± SEM. Two-tailed unpaired t-test (**i**), two-way ANOVA with Bonferroni's multiple comparisons test(**j**). $^*p < 0.05$, $^{**}p < 0.01$, $^{***}p < 0.001$. Source data are provided as a Source Data file.

FigureS4

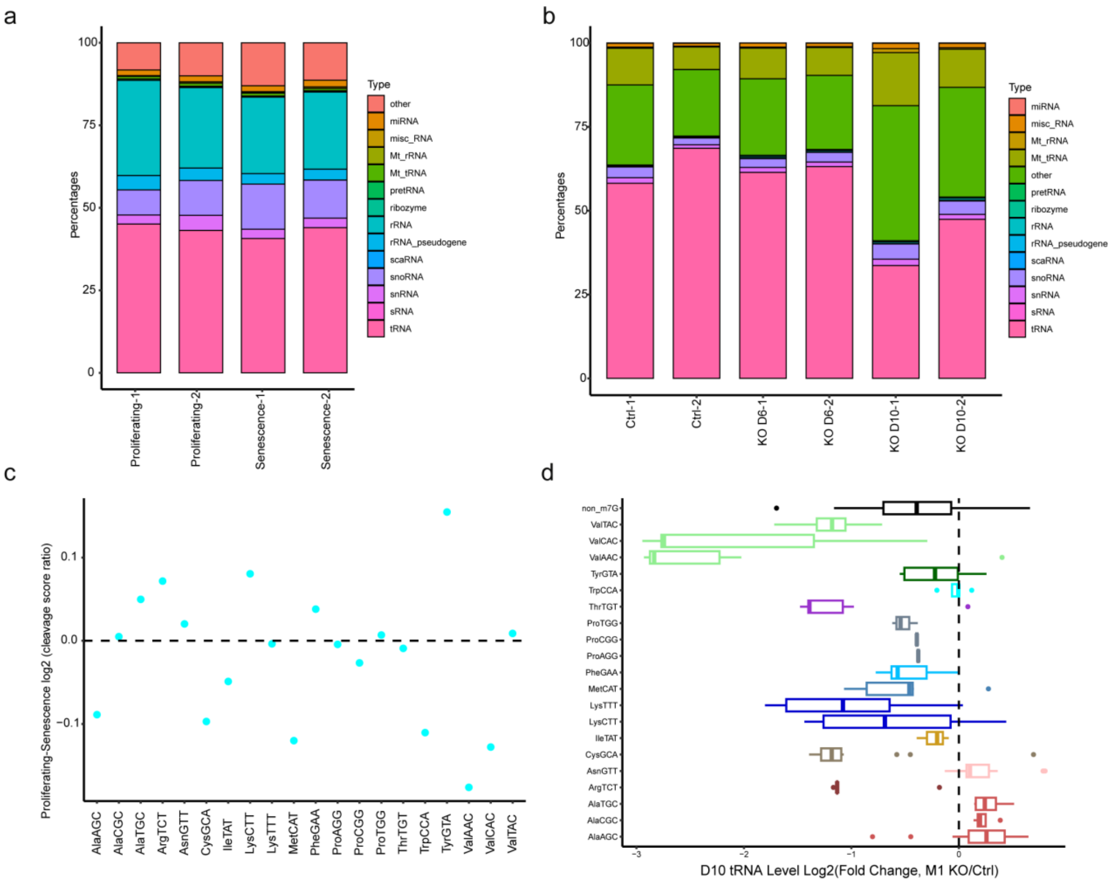

FigS4. METTL1 Deficiency Downregulates a Group of m7G-tRNAs

a The proportion of small RNAs mapping in tRNA-seq samples from senescence groups was shown. **b** The proportion of small RNAs mapping in tRNA-seq samples from METTL1 knockout (KO) groups was shown. **c** Ratios of cleavage scores in young and senescent cells were characterized. Cleavage Score Ratio = Cleavage Score (Sample 1) / Cleavage Score (Sample 2). **d** Differentially expressed m7G-tRNAs in control/METTL1 KO (Day 10) cells were showed. All tRNA genes of the same tRNA type were combined to calculate their transcript abundance. Source data are provided as a Source Data file.

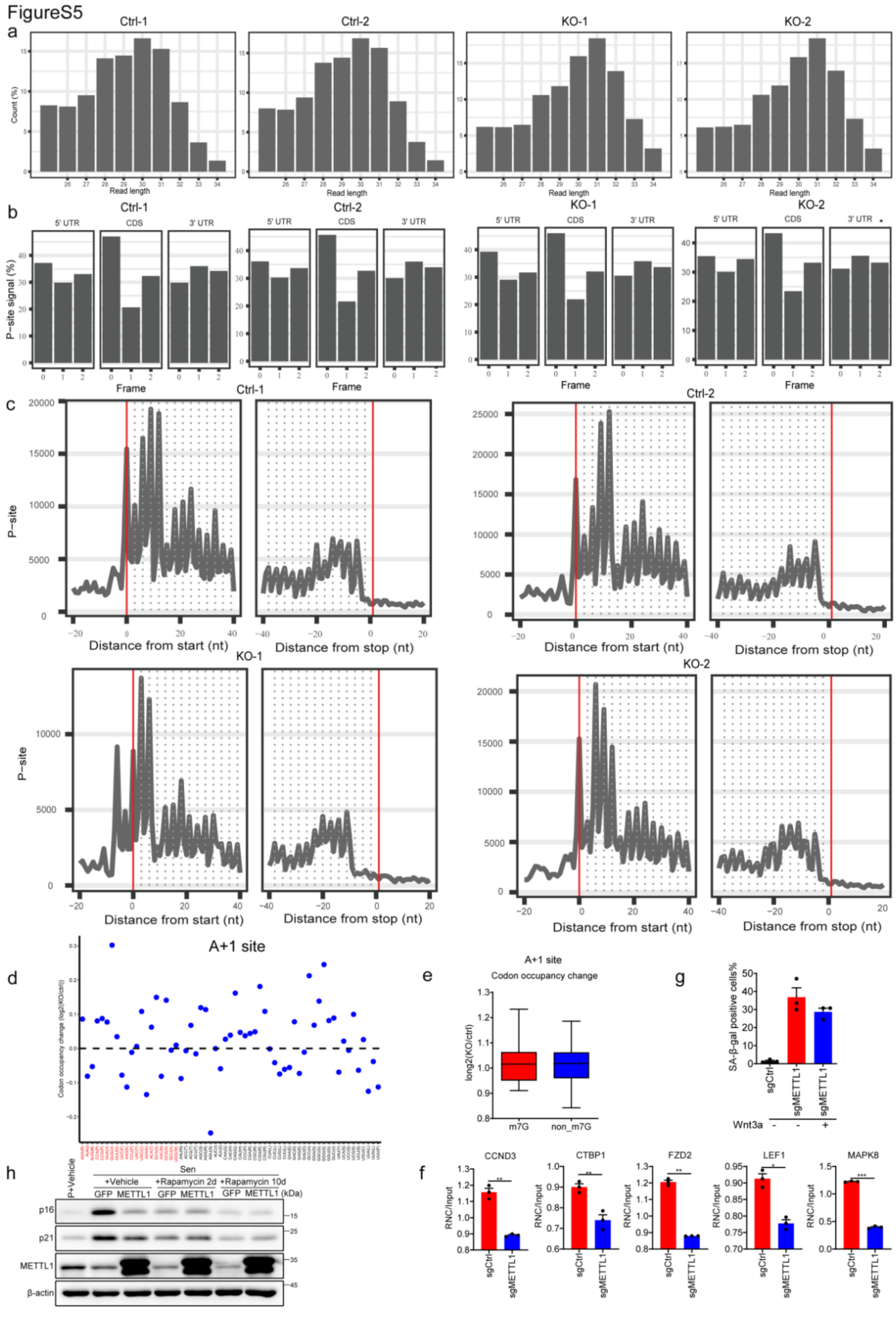

FigS5.Ribo-Seq Uncover Codon Usage upon Mettl1 Depletion

a-c Characteristics of ribosome footprinting. (**a**) Distribution of the read length. (**b**) The percentage of P-sites falling in three possible translation reading frames for 5' UTRs, CDSs, and 3' UTRs in ribosome footprinting data. (**c**) The meta-

profile was generated based on the mapping of P-sites around the start and stop codons of annotated CDSs. **d** Ribosome occupancy at individual codons at the A+1 site. Plots represent the relative ribosome footprinting signals from Mettl1 KO and control cells. The codons are separated into m7G-tRNA decoding codons(red) and non-m7G-tRNA decoding codons(black). **e** Mann-Whitney U test on the the ribosome occupancy at individual codons in A +1 sites from m7G-tRNA and non-m7G-tRNA. The ribosome footprinting signals from METTL1 sgRNA and scramble control were presented. **f** Translation ratios of Wnt signaling pathway proteins were assessed using RNC-qPCR. **g** SA-β-gal staining was performed on WNT3a-conditioned medium treated cells as indicated. **h** Protein levels of p16 and p21 in METTL1 overexpressing cells with or without rapamycin(10nM) treatment were conducted. Representative result was shown, three repeats demonstrate similar results (**h**). All the assays were biologically repeated three times(**f,g**). All data were presented as the mean ± SEM. Two-tailed unpaired t-test (f), one-way ANOVA with Bonferroni's multiple comparisons test(g). $*p < 0.05$, $**p < 0.01$, $***p < 0.001$. Source data are provided as a Source Data file.

FigureS6

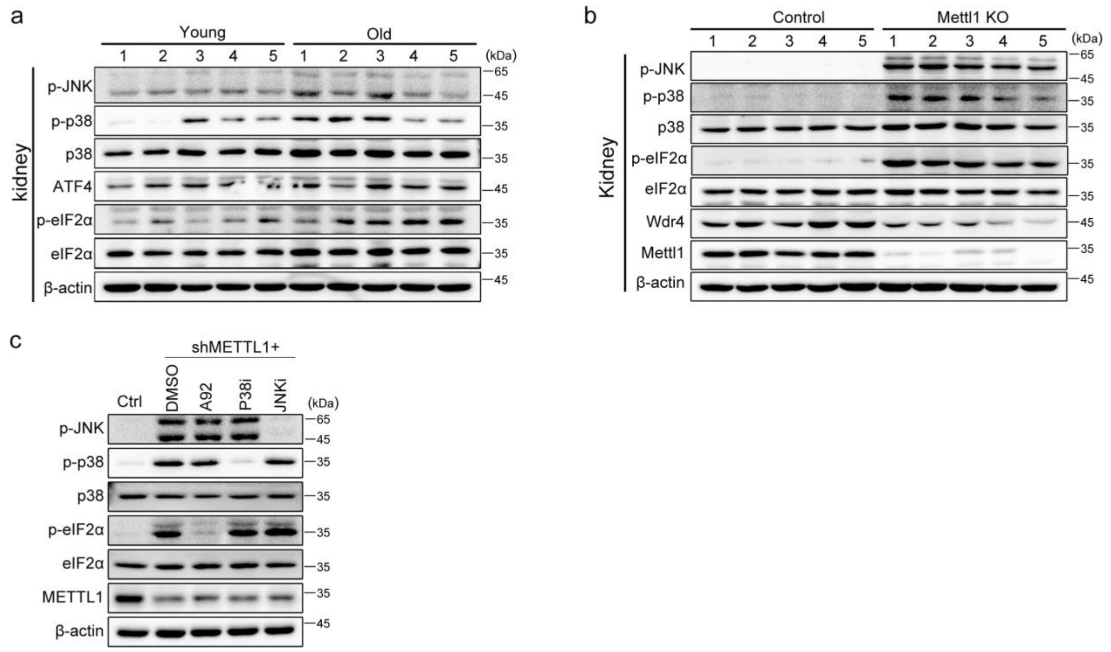

FigS6.METTL1 Deficiency Elicits Ribotoxic Stress Response and Integrated Stress Response a ISR and RSR response-relevant proteins were detected by Western blotting using kidney tissues from young/old mouse(**a**) (n=5 mice per group, 2 female and 3 male) and Mettl1 KO/ control mice(**b**) (n=5 mice per group, 3 female and 2 male) kidney tissues. **c** Validation of the effect of GCN2 and p38 inhibitors on METTL1-depleted cells was performed by western blotting to detect eIF2α and p38 phosphorylation. All WB results were performed three times, the representative result was shown.

FigureS7

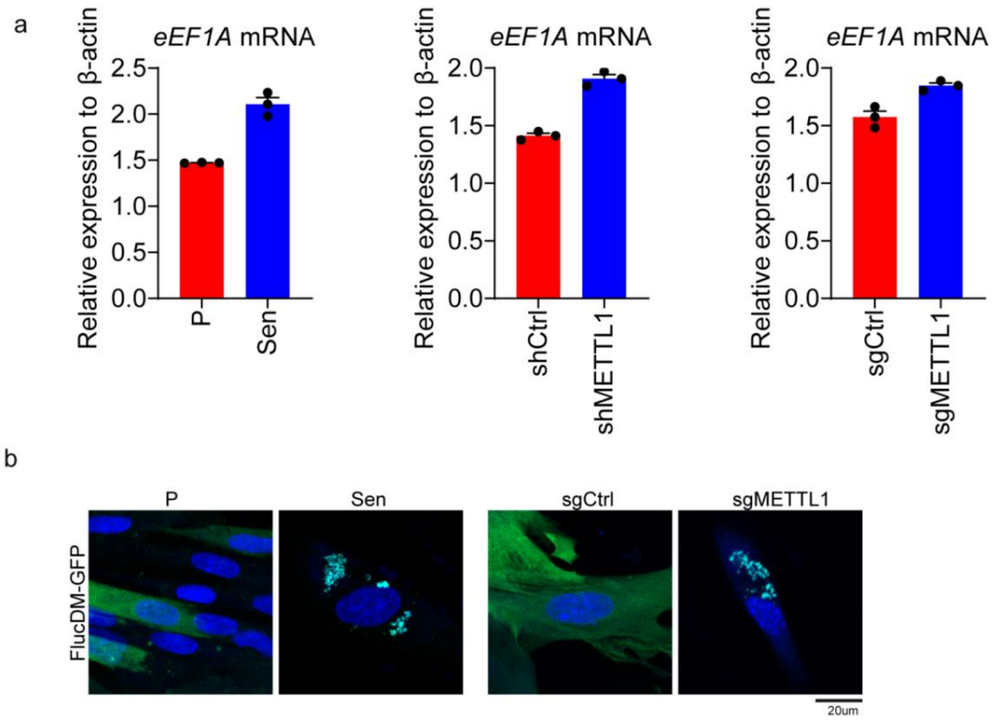

FigS7. RTD is Involved in the Senescence Caused by METTL1 Deficiency a eEF1A level was detected by RT-qPCR with samples of replicative senescence, and METTL1 depleted IMR90 cells by shRNAs and sgRNAs. Three repeats were conducted, P values were calculated using a two-tailed Student's T-test. Data was presented as mean ± SEM. b Represent images of FlucDM-GFP expressing cells are showed. Source data are provided as a Source Data file.

FigureS8

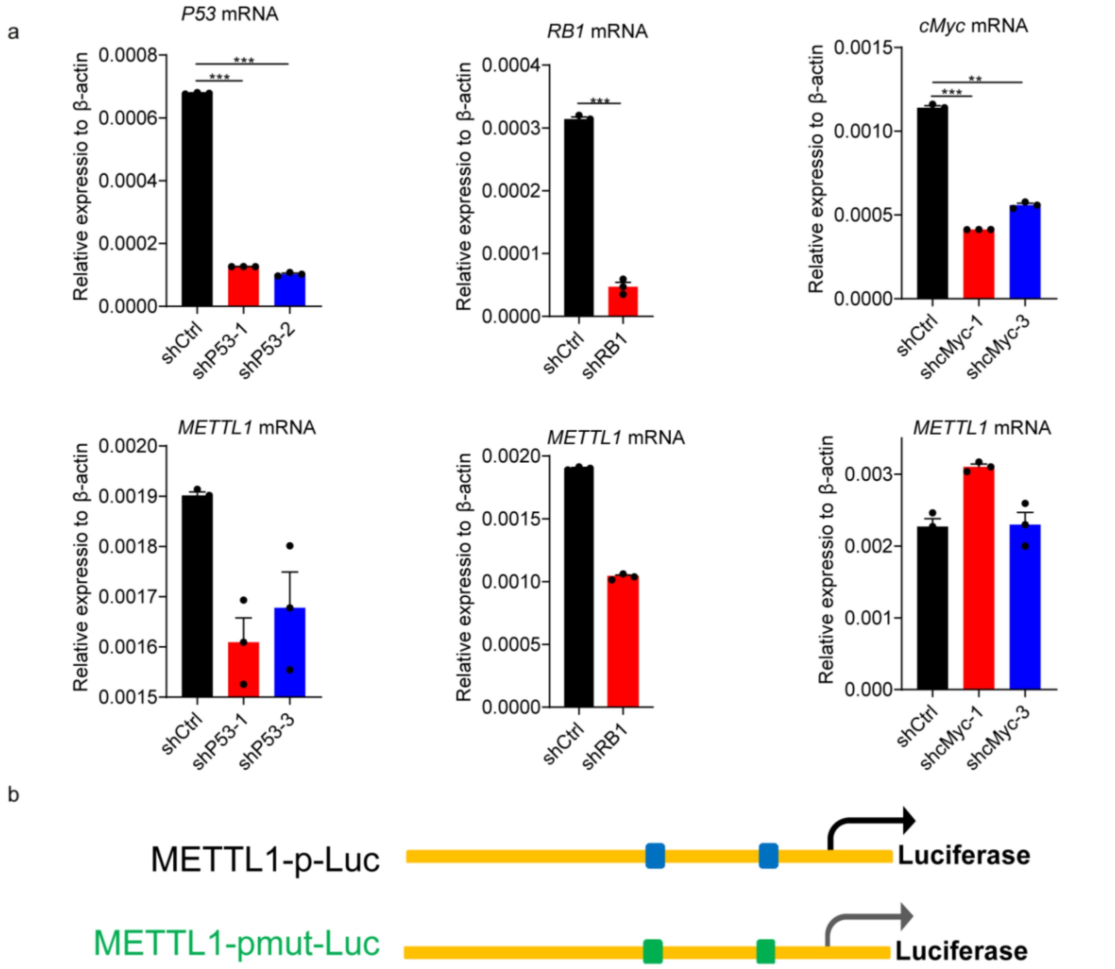

FigS8. SP1 Regulates METTL1 and WDR4 Expression

a METTL1 level were quantified using RT-qPCR in IMR90 cells with short hairpin RNAs (shRNAs) targeting p53, RB1, and cMyc. Three biologically repeats were conducted. P values were calculated using a two-tailed Student's T-test. Data is presented as mean ± SEM. $*p < 0.05$, $**p < 0.01$, $***p < 0.001$. **b** Schematic diagram of dual luciferase reporter gene assay. Source data are provided as a Source Data file.

Supplementary table 1. shRNA, sgRNA, siRNA targeting sequence and cloning primers

shMETTL1 1#	CCCACATTTCAAGCGGACAAA
shMETTL1 3#	GGTGTATACCATAACCGATGT
shSP1 1#	CCACTCCTTCAGCCCTTATTA
shSP1 2#	GGCAGATCTGCAGTCCATTAA
shP53 1#	CACCATCCACTACAACTACAT
shP53 2#	GTCCAGATGAAGCTCCCAGAA
shRB1	CAGAGATCGTGTATTGAGATT
shcMyc 1#	ATGTCAAGAGGCGAACACAC
shcMyc 2#	GATGAGGAAGAAATCGATG
sgMETTL1 2#	GCAGCGTGCTCACAGCAATC
sgMETTL1 4#	ACATAGGCTGTGGCTATGG
siZAKα(NM_016653) 1#	CCCATTAAGTATCAACAGA
siZAKα(NM_016653) 2#	CAGACAGAAGCAGGAACAA
METTL1-F	TCGAGACTAGTGTTTATGGCAGCCGAGACTCGGAA
METTL1-R	GGCGGGGAAGGCGAAGAACATCTTTGTCAGCT
METTL1-AFPA mut-F1	TCGAGACTAGTGTTTATGGCAGCCGAGACTCGGAA
METTL1-AFPA mut-F2	TTCGCCTTCCCCGCCCCACATTTCAAGCGGACAAA
METTL1-AFPA mut-R1	ATCCTTGTAATCGTTGTGACCAGGCAGGCTGGTTT
METTL1-AFPA mut-R2	GGCGGGGAAGGCGAAGAACATCTTTGTCAGCT
eEF1A-F	CTCTAGAGTTTAAACATGGGAAAGGAAAAGACTCAT
eEF1A-R	CTTGTAATCGTTTACGTATTTAGCCTTCTGAGCTTTCT
METTL1-1K-promoter-F	GTACCGAGCTCTTACGCGTACGATGCCTGTCCCCGCACCCA
METTL1-1K-promoter-R	GATCGCAGATCTCGAGACCAAATCCACGTGGAGGCG
METTL1-1K-promoter-mut-1-F	GGGTCTTTAAATTAATATTCCTGCCACCAACGCGCATGGCT
METTL1-1K-promoter-mut-1-R	GGAATATTAATTTAAAGACCCAAGAGCAAATTCAGCT
METTL1-1K-promoter-mut-2-F	TTAACTATAGGGTCCGAGACCAGGAGTAAGGG
METTL1-1K-promoter-mut-2-R	GTCTCGGACCCTATAGTTAAGTCTGACTCTTGTGGTCCCGT

Supplementary table 2. Antibodies

Antibodies	SOURCE	IDENTIFIER
Rabbit polyclonal anti-Mettl1	Proteintech	Cat# 14994-1-AP
Rabbit polyclonal anti-beta-Actin	ABclonal	Cat# AC026
Mouse monoclonal 7-methylguanosine (m7G)	MBL life science	Cat# RN017M
Rabbit monoclonal anti-Wdr4	Abcam	Cat# ab169526(for human cells)
Rabbit monoclonal anti-Wdr4	Siganlway antibody	Cat# 42846(for mouse cells)
Rabbit polyclonal anti-LMNB1	Abcam	Cat# ab16048
Rabbit polyclonal anti-Ezh2	Cell Signaling Technology	Cat# 5246T
Rabbit polyclonal anti-p16 INK4A	Proteintech	Cat# 10883-1-AP(for human cells)
Rabbit polyclonal anti-p16 INK4A	Siganlway antibody	Cat# 41296(for mouse cells)
Rabbit polyclonal anti-p21 Waf1/Cip1/CDKN1A	Proteintech	Cat# 10355-1-AP
Rabbit polyclonal anti-eIF2α	ABclonal	Cat# A0764
Rabbit polyclonal anti-Phospho-eIF2α (Ser51)	Cell Signaling Technology	Cat# 3398
Rabbit polyclonal anti-ATF4	Proteintech	Cat# 10835-1-AP
Rabbit polyclonal anti-p38 MAPK	ABclonal	Cat# A14401
Rabbit polyclonal anti-Phospho-p38 MAPK (Thr180/Tyr182)	Cell Signaling Technology	Cat# 4511S
Rabbit polyclonal anti-Phospho-SAPK/JNK (Thr183/Tyr185)	Cell Signaling Technology	Cat# 4668
Rabbit polyclonal anti-RPL8	ABclonal	Cat# A10042
Rabbit polyclonal anti-RPS20	Proteintech	Cat# 15692-1-AP
Mouse monoclonal anti-puromycine	Millipore	Cat# MABE343
Rabbit polyclonal anti-β-Catenin	Proteintech	Cat# 51067-2-AP
Mouse monoclonal anti-Digoxigenin (DIG)	MBL life science	Cat# M227-3
Anti-rabbit IgG HRP-linked Antibody	Cell Signaling Technology	Cat# 7074S

Anti-mouse IgG HRP-linked Antibody	Cell Signaling Technology	Cat# 7076S
Rabbit polyclonal anti-eEF1A	Proteintech	Cat# 11402-1-AP
Rabbit polyclonal anti-SP1	Proteintech	Cat# 21962-1-AP
Normal Rabbit IgG	Cell Signaling Technology	Cat# 2729S

Supplementary table 3. qPCR primers for human, mouse, Drosophila

**Human**	
actin-F	AGAAAATCTGGCACCACACC
actin-R	AGAGGCGTACAGGGATAGCA
p16 INK4A-F	CCAACGCACCGAATAGTTACG
p16 INK4A-R	CCATCATCATGACCTGGATCG
METTL1-F	CGCTACCCTGTGAAGCCAGA
METTL1-R	AGCGGTGACAGTTCCACTAA
WDR4-F	CTGGGCTGGCACATGTGTAT
WDR4-R	GCTGTCATCATCACTGCTTGC
IL1A-F	TGGTAGTAGCAACCAACGGGA
IL1A-R	ACTTTGATTGAGGGCGTCATTC
IL1B-F	CCACCTCCAGGGACAGGATA
IL1B-R	TGGGATCTACACTCTCCAGC
CXCL5-F	ACAGACCACGCAAGGAGTTC
CXCL5-R	TCCTTGTTTCCACCGTCCAA
CXCL8-F	TGGAGAAGTTTTTGAAGAGGGCT
CXCL8-R	CAACAGACCCACACAATACATGA
IL6-F	TTCTCCACAAGCGCCTTC
IL6-R	AGAGGTGAGTGGCTGTCTGT
WNT2-F	CCGAGGTCAACTCTTCATGGT
WNT2-R	CCTGGCACATTATCGCACAT
CCND3-F	GCTTACTGGATGCTGGAGGTAT
CCND3-R	TGGGGACGCAAGACAGGTA
CTBP1-F	CAGAGCCATCACAGGCCG
CTBP1-R	CCTATAGGCAGCCCCATTGAG
FZD2-F	GTGCCATCCTATCTCAGCTACA
FZD2-R	CTGCATGTCTACCAAGTACGTG
LEF1-F	TGCCAAATATGAATAACGACCCA
LEF1-R	GAGAAAAGTGCTCGTCACTGT
MAPK8-F	CAGCCCTCTCCTTTAGGTGC
MAPK8-R	GCTGCTGCTTCTAGACTGCT
eEF1A-F	GGACACGTAGATTCGGGCAA
eEF1A-R	AGGAGCCCTTTCCCATCTCA
**Mouse**	
actin-F	CAGAAGGAGATTACTGCTCTGGCT

actin-R	TACTCCTGCTTGCTGATCCACATC
Mettl1-F	ACGCCATGAAACACCTTCCT
Mettl1-R	TACACCAGGCCCCCGACT
p16 INK4A-F	CCGCTGCAGACAGACTGG
p16 INK4A-R	CCATCATCATCACCTGAATCG
**Drosophila**	
Wuho-QF	CAATTTCGTTCGCGGAACCC
Wuho-QR	TACACGATTCCTGCGACTGG
CG4045-QF	GAAGAGGAGGCCAATGCTGA
CG4045-QR	TCCTAAAGGCGCCGGAAAAT
Rpl18-QF	GTTGCTCCAAACCCTCCA
Rpl18-QR	GATCCGTCTAACACCTCCC

Supplementary table 4. Northern blotting probes

snoU6	TGGAACGCTTCACGAATTTG
Val-CAC	GACCTTTCGCGTGTGAGGCGAAC
Val-AAC	TGTTTCCGCCCGGTTTCGAA
Val-TAC	TGGTTCCACTGGGGCTCGAA
Pro-CGG-1	GGGACCTCTCGCACCCGAAGCGAGAA
Pro-CGG-2	CCGAAGCGAGAATCATACCCCTAG
Cys-GCA-1	GGGACCTCTTGATCTGCAGTCAAATG
Cys-GCA-2	AGGGGGCACCTGGATTTGAACC
Thr-AGT	AGGCCTCGCTGGGATTCGAACCC

Supplementary table 5. ChIP-qPCR primers

METTL1-ChIP-F1	CAGCCCAGCCCAGCTGAA
METTL1-ChIP-R1	ATCCCAGTCCGGGGTTTCTCT
METTL1-ChIP-F2	GGACCACAAGAGTCAGACCG
METTL1-ChIP-R2	CGCCTTCTACAGTGGCATCA
METTL1-ChIP-F3	GTGTCCCTGTGGAACGAGC
METTL1-ChIP-R3	TCCACCTCCAGGAAATCGAAC
METTL1-ChIP-F4	CCACGAAGTGACCATTTCTCCGC
METTL1-ChIP-R4	GTCTTTTCTCCCGTAGGCCCTG
METTL1-ChIP-F5	GTAGCCGGGTATGGTAGCGCA
METTL1-ChIP-R5	GGCAGGAAATAAAACTCAGGCAGG
