# Supplementary material for: Perturbation of METTL1-mediated tRNA N7- methylguanosine modification induces senescence and aging
Source: Nat Commun. 2024 Jul 8;15:5713. doi: 10.1038/s41467-024-49796-8 (PMC11231295; doi:10.1038/s41467-024-49796-8)
Supplement: Supplementary file 3 — Description of Additional Supplementary Files [file 41467_2024_49796_MOESM3_ESM.docx]

**Description of Additional Supplementary Files**

**File name: Supplementary Data 1**

Description: Proteomics analysis for proliferating and senescent IMR90 cells.

**File name: Supplementary Data 2**

Description: Transcriptome data for METTL1 Knockout in IMR90

cells.

**File name: Supplementary Data 3**

Description: tRNA m7G cleavage scores calculated from TRAC-

Seq data.

**File name: Supplementary Data 4**

Description: Read counts of tRNA transcript in TRAC-Seq.

**File name: Supplementary Data 5**

Description: Codon occupancy at individual codon of A and A+1 sites. Translation efficiency of mRNAs from METTL1 KO IMR90

cells.

**File name: Supplementary Data 6**

Description: Reagents and plasmids were listed in the files.

**File name: Supplementary Data 7**

Description: HPLC-MS data for m7G detection in Fig1F- 1G, Fig3B, FigS2E and FigS2G.
